# Squamous cell carcinoma of verrucous type in the setting of prior pyoderma gangrenosum: a case report

**DOI:** 10.1186/s13256-022-03412-9

**Published:** 2022-05-23

**Authors:** Amy Morrison, Carlene Waters-Hollingsworth, G. Mabel Gamboa

**Affiliations:** 1 University of Central Florida College of Medicine, Orlando, USA; 2 Orlando VA Medical Center, Orlando, USA

**Keywords:** Case report, Squamous cell carcinoma, Pyoderma gangrenosum

## Abstract

**Introduction:**

Pyoderma gangrenosum and cutaneous squamous cell carcinoma are two conditions well reported in the literature and may exist concurrently in the same patient. In fact, there have been reports of misdiagnosis of one for the other. The two conditions occurring in the same location, however, has not yet been reported.

**Case description:**

We report the case of a 57-year-old Caucasian male with history of pyoderma gangrenosum of the right lateral tibial area who developed squamous cell carcinoma of verrucous type at the same site. Excisional surgery was considered to treat this patient, but he was ultimately managed with radiation therapy to the affected area due to the size of the lesion and the risk of triggering proliferation of the pyoderma gangrenosum.

**Conclusions:**

We hope that this case report will add to the literature of both conditions, show a unique presentation of both conditions, and emphasize the inclusion of both pyoderma gangrenosum and squamous cell carcinoma when developing a differential diagnosis of a chronic, nonhealing wound.

## Introduction

Squamous cell carcinoma is the second most common form of skin cancer, with an incidence of 1.8 million per year, and is increasing each year [1]. Squamous cell carcinoma can present on areas of the skin that are prone to photodamage and may grossly appear as scaly papules or plaques but may have a variety of different textures. Pyoderma gangrenosum is a neutrophilic dermatosis that has an incidence of 3–10 per million people per year, and often exists concurrently with autoimmune disorders or as an inherited autoimmune syndrome [2]. Patients most commonly present with a large, painful, ulcerated skin lesion. Both conditions are very well characterized in the literature and might be misdiagnosed for each other. However, while both conditions may occur concurrently in the same patient, they have not yet been reported in the literature at the same site. We report a patient with squamous cell carcinoma of verrucous type that developed at the site of prior pyoderma gangrenosum.

## Case description

A 57-year-old Caucasian male with history of Crohn’s disease, colon cancer, and bilateral deep vein thrombosis (DVT) was referred to the Plastic Surgery Clinic from a dermatologist for a chronic, nonhealing skin lesion over the right lateral tibia, present for the past 13 years. The lesion was previously diagnosed as biopsy-proven pyoderma gangrenosum, being refractory to multiple treatment options, and passing through phases of waxing and waning severity. He is currently taking Humira, which has helped debulk the lesions. Past surgical history includes hydrocele excision, right ventral hernia repair with mesh, left groin lipoma excision, inferior vena cava (IVC) filter, colon resection, splenectomy, cholecystectomy, and leg DVT removal.

**Fig. 1:**
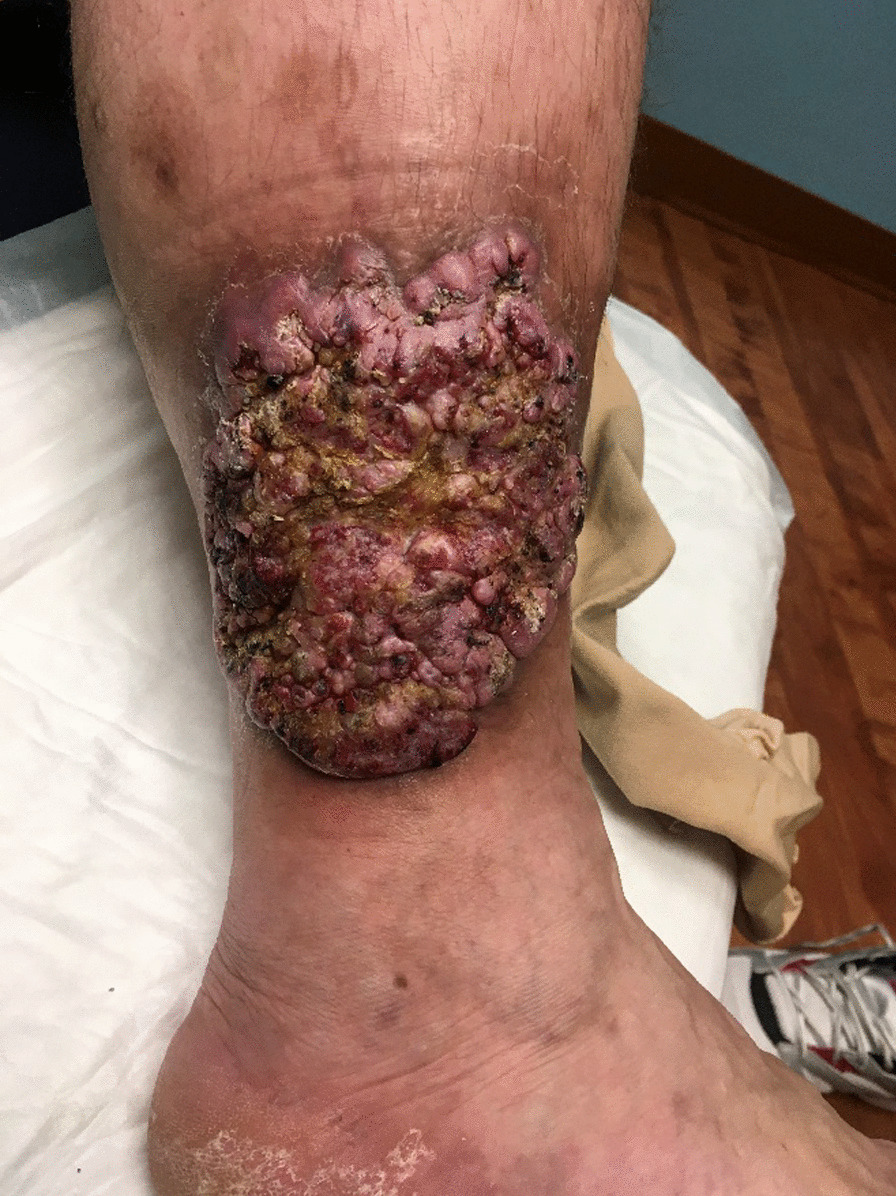
Gross imaging from June 2021, showing a raised, exophytic, fungating, irregularly shaped lesion on the right lateral tibia

Examination of the lesion revealed an irregularly shaped, raised, fungating, exophytic lesion with no bleeding or infection. The entire lesion measures 12.0 cm × 12.0 cm × 1 cm as shown in Fig. 1. Other skin changes seen on physical examination include bilateral hyperpigmentation of lower extremities, consistent with evidence of venous stasis. His Crohn’s disease is currently being managed, and on examination the abdomen was soft, nontender, with normoactive bowel sounds in all four quadrants and no evidence of organomegaly.

The patient has no history of tobacco use or recreational drug use. He quit drinking alcohol 30 years ago.

A punch biopsy of the right lateral tibia was performed four years prior, revealing lobular vascular proliferation within the dermis with associated hemosiderin and spongiosis of the epidermis consistent with stasis dermatitis. More recently, at the time of presentation, punch biopsies of the same area revealed invasive squamous cell carcinoma of verrucous type.

### Imaging findings

An extremity venous limited ultrasound performed the month prior showed new partial compressibility of the proximal femoral vein and partial compressibility of the popliteal vein stable from 2019, unable to distinguish between acute nonocclusive thrombus within the proximal femoral vein versus manifestations of chronic DVT. X-ray of the right tibia/fibula, also performed the month prior, showed no acute osseus injury or aggressive osseous destruction of the tibia/fibula. Positron emission tomography/computed tomography from the patient's skull base to thigh performed month of presentation showed a large, intensely hypermetabolic lesion along the skin of the right calf, measuring up to 105 mm, with no clear delineation between the two suspected process, pyoderma gangrenosum and squamous cell carcinoma. Imaging findings displayed hypermetabolic, enlarged right inguinal and external iliac lymph nodes, noted to be a non-specific finding in this context. It was noted that chronic wounds or metastasis may display this pattern, size, and degree of uptake. No evidence of distant metastasis was found.

## Management

Ultimately, the patient was not managed surgically. Surgical management of the squamous cell carcinoma would be challenging due to the potential of triggering proliferation of the pyoderma gangrenosum. Creation of a flap or skin graft large enough for closure of the lesion has additional challenges that were considered. He was referred to radiation oncology for treatment of the lesion. He continues to take Humira and completed daily radiation therapy of the right lateral tibia area two months later.

## Discussion

No cases of squamous cell carcinoma of the skin at the same site as pyoderma gangrenosum have been reported in literature to date. One case of pyoderma gangrenosum and squamous cell carcinoma was reported, occurring in the same patient; in a patient with metastatic squamous cell carcinoma, Tsibris *et al*. reported an instance of pembrolizumab-triggered pyoderma gangrenosum on the back and the right shin [3]. However, this was thought to be associated with the pembrolizumab treatment rather than the metastatic disease. Three cases of pyoderma gangrenosum were initially diagnosed as squamous cell carcinoma: the first, located on the left dorsal hand, reported by Wolfe *et al*. in 2012 [4], the second, a reported pyoderma gangrenosum on the trunk that had been misdiagnosed and treated for squamous cell carcinoma in 2015 reported by Čuk Radović *et al*. [5], and a third, located on the trunk, reported by Gonzalez-Sabin *et al*. in 2019 [6]. Lastly, and conversely, a case of squamous cell carcinoma resembling pyoderma gangrenosum was reported by Emmert *et al*. in 2015. The lesion was located on the right thumb [7]. These findings are summarized in Table 1.

**Table 1:** Summary of pyoderma gangrenosum and squamous cell carcinoma case review

Author	Title	Source	Year	Summary of case
Tsibris *et al*.	Pembrolizumab-associated pyoderma gangrenosum in a patient with metastatic squamous cell carcinoma	*Dermatology Online Journal*	2021	Pyoderma gangrenosum developed on the back and right shin of a patient being treated with Pembrolizumab for metastatic squamous cell carcinoma.
Wolfe *et al*.	Atypical pyoderma gangrenosum of the dorsal hand mimicking squamous cell carcinoma	*Journal of Hand Surgery*	2012	Pyoderma gangrenosum was initially misdiagnosed as squamous cell carcinoma on the left dorsal hand.
Čuk Radović *et al*.	Advanced pyoderma gangrenosum previously treated as squamous cell carcinoma	*Acta Dermatovenerol Croat*	2015	Pyoderma gangrenosum was initially misdiagnosed as squamous cell carcinoma on the trunk.
Gonzalez-Sabin *et al*.	Pioderma gangrenoso simulando un carcinoma epidermoide	*Actas Dermo-Sifiliográficas*	2019	Pyoderma gangrenosum was initially misdiagnosed as squamous cell carcinoma on the trunk.
Emmert *et al*.	Squamous-cell carcinoma resembling pyoderma gangrenosum	*New England Journal of Medicine*	2015	A case of squamous cell carcinoma resembling pyoderma gangrenosum was found on the right thumb.

Additionally, verrucous types of squamous cell carcinoma most commonly occur on the head and neck rather than the lower leg, although instances on the lower extremities have been reported. One case of verrucous-type squamous cell carcinoma arose at a chronic neuropathic ulcer of a patient with leprosy, hypothesized to be due to chronic inflammation in the wound [8]. It is reasonable to consider that, in this patient, the longstanding inflammation from pyoderma gangrenosum provoked formation of squamous cell carcinoma.

Finally, it is important to be aware of the risk that surgical treatments may pose to pyoderma gangrenosum. It is possible that surgery may trigger proliferation of pyoderma gangrenosum or increase risk of recurrence and thus increase the severity of a patient’s condition. The patient described is being managed with radiation therapy for this reason.

Strengths of this case report include the unique nature of the case. This patient highlights a presentation of two well-known conditions that has not been described before. It emphasizes the need to include both conditions in the differential of an area of chronic inflammation or a nonhealing wound. Limitations include that specific interventions prior to the Humira are unknown, and the patient’s family history is unknown.

## Conclusion

It is important to include both pyoderma gangrenosum and squamous cell carcinoma in the differential diagnosis when evaluating nonhealing skin lesions. Furthermore, any patient with pyoderma gangrenosum or any chronic nonhealing wound should undergo continuous surveillance due to the possibility of transformation to malignancy.

## Data Availability

Data sharing is not applicable to this article as no datasets were generated or analyzed for this case report.

## Appendix

See Fig. 1 and Table 1.

## References

[1] Skin Cancer Facts & Statistics. The Skin Cancer Foundation. https://www.skincancer.org/skin-cancer-information/skin-cancer-facts/ (2021). Accessed July 10 2021.

[2] Ashchyan HJ, Nelson CA, Stephen S, James WD, Micheletti RG, Rosenbach M. Neutrophilic dermatoses: pyoderma gangrenosum and other bowel- and arthritis-associated neutrophilic dermatoses. J Am Acad Dermatol. 2018;79(6):1009–22. 10.1016/j.jaad.2017.11.063 29653213

[3] Tsibris H, Lian C, Ho A. Pembrolizumab-associated pyoderma gangrenosum in a patient with metastatic squamous cell carcinoma. Dermatol Online J. 2021. 10.5070/d3274053158. 33999581

[4] Wolfe CM, Green WH, Cognetta AB, Baniahmad O, Hatfield HK. Atypical pyoderma gangrenosum of the dorsal hand mimicking squamous cell carcinoma. J Hand Surg. 2012;37(9):1835–8. 10.1016/j.jhsa.2012.06.019. 22854258

[5] Čuk Radović T, Kostović K, Radoš J, Paštar Z, Pavliša G, Marinović B. Advanced pyoderma gangrenosum previously treated as squamous cell carcinoma. Acta Dermatovenerol Croat. 2015;23(3):208–12. 26476906

[6] González-Sabín M, Rodríguez-Díaz E, Gonzalvo-Rodríguez P, Astola-Hidalgo I. Pioderma gangrenoso simulando un carcinoma epidermoide. Actas Dermosifiliogr. 2019;110(5):400–2. 10.1016/j.ad.2018.02.022 30072023

[7] Emmert S, Schön MP. Squamous-cell carcinoma resembling pyoderma gangrenosum. N Engl J Med. 2015. 10.1056/nejmicm1410236. 26222581

[8] Khullar G, Mittal S, Sharma S. Verrucous carcinoma on the foot arising in a chronic neuropathic ulcer of leprosy. Australas J Dermatol. 2019;60(3):245–6. 10.1111/ajd.13020. 30841011

